# Basic Experimental and Clinical Advances in the Mechanisms Underlying Abnormal Pregnancy Outcomes

**DOI:** 10.1155/2013/327638

**Published:** 2013-02-11

**Authors:** Timothy R. H. Regnault, Mark J. Nijland, Helen Budge, Janna L. Morrison

**Affiliations:** ^1^Departments of Obstetrics and Gynaecology/Physiology and Pharmacology, Children's Health Research Institute, Western University, London, ON, Canada N6A 5C; ^2^Center for Pregnancy and Newborn Research, Department of Obstetrics and Gynecology, School of Medicine, University of Texas Health Science Center at San Antonio, San Antonio, TX 78229-3309, USA; ^3^Academic Child Health, School of Clinical Sciences, University of Nottingham, Nottingham, UK; ^4^Early Origins of Adult Health Research Group, School of Pharmacy and Medical Sciences, Sansom Institute for Health Research, University of South Australia, GPO Box 2471, Adelaide, SA 5001, Australia

Development and growth of the embryo and fetus are complex processes inextricably linked to the ability of the individual to reach childhood developmental milestones. Insults or stimuli to which the embryo or fetus responds during critical periods of intrauterine growth and development are now understood to alter the genetically dictated growth trajectory of the fetus and reprogram the newborn's long-term development and physiological path. Basic experimental and clinical human studies demonstrate that intrauterine growth restriction may result from *in utero* insults such as uteroplacental insufficiency and/or hypoxia, preeclampsia, preterm labor, and imbalanced nutrition. The fetal responses to these insults manifest as a predisposition to the development of the metabolic syndrome in postnatal life. The “thrifty phenotype hypothesis” has been proposed to explain the mechanism by which *in utero* responsive adaptations set in motion the series of developmental and physiological adaptations to maximize the chances of *in utero* survival [1–3]. While intrauterine growth restriction resulting in low birth weight is recognized as a major risk factor for noncommunicable diseases associated with the metabolic syndrome, it is now apparent that both ends of the birth weight spectrum are associated with having an increased risk of adult-onset disease in later life [4, 5]. This further highlights the necessity to understand both optimal growth patterns that result in optimal birth weight and the critically important role that they play in setting the stage for health and the risk of disease in postnatal life. In this special issue, we have gathered a number of excellent reviews, each focussing upon different facets of how an altered *in utero* environment results in adaptations in fetal organ systems, expanding our understanding of how perturbations at early stages of development may have long-term consequences.

The placenta represents not only the junction between the maternal and fetal circulatory systems, but also plays an important role in regulating fetal nutritional supply. The regulation of placental nutrient transport is multifactorial. The review by S. Lager and T. L. Powell in this issue focuses on factors regulating placental glucose, amino acid, and fatty acid transporters. Particularly intriguing is the concept that the placenta plays a major role in integrating maternal and fetal signals to regulate overall nutrient supply, transport, and thus fetal growth. Keeping with placental function, L. Avagliano et al. discuss the mechanisms controlling placental amino acid transport in the context of placental insufficiency that results in IUGR. Their review maintains a specific emphasis on the human condition and the impact changes in maternal amino acid concentrations have on placental transport and metabolism. The theme of the central role played by the placenta in fetal growth and development is highlighted in the article by D. T. Yates et al. which examines the topic of placental insufficiency and reduced newborn muscle mass. In their review, they probe the contribution of reduced fetal oxygen tension during late gestation to modulating circulating catecholamines, as chronic hypercatecholaminemia reduces both fetal muscle development and growth. They highlight that developmental programming of skeletal muscle adrenergic receptors may partially explain how the metabolic and endocrine phenotype of IUGR offspring leads to differential nutrient utilization, linking skeletal muscle development and later life disease risk. Adipose tissue has also been identified as being highly plastic and vulnerable to changes in the *in utero* environment, leading to alterations in postnatal metabolism. Various adipose depots appear to be sensitive to the *in utero* environment, such that altered *in utero* development may provide important linkages to aspects of the metabolic syndrome in adult life. O. Sarr et al. discuss how IUGR fetuses display increased lipogenic and adipogenic capacity as offspring and they review the roles factors such as fetal hypoleptinemia, altered glucocorticoid signaling, and chromatin remodeling may subsequently contribute to an increased later life obesity risk.

An emerging theme in the field of developmental programming stems from observations that different insults *in utero* result in similar adverse postnatal phenotypes associated with the metabolic syndrome. The idea of a common factor acting across the spectrum of *in utero* insults has been suggested to explain this phenomenon. Indeed, oxidative stress and activation of inflammatory pathways have now been proposed as key modulators of fetal growth [6, 7]. Oxidative stress in the fetal compartment can be generated by several conditions, such as prenatal hypoxia, maternal under- and overnutrition, and inappropriate exposure to glucocorticoids. In their review, L. P. Thompson and Y. Al-Hasan discuss possible origins of developmental reprogramming as a result of *in utero* oxidative stress. They highlight the important role that oxidant molecules have as signalling factors in developmental programming and propose that these effects are mediated by epigenetic mechanisms. In addition to oxidative stress pathways, inflammatory complications that impact on proinflammatory cytokines such as IL-1, IL-6, and TNF-*α*, as well as increased concentrations of glucocorticoids also have major roles in the onset of altered growth patterns *in utero* [8, 9]. R. E. Fisher et al. review the offspring's susceptibility to a range of metabolic disorders while focusing on the impact of activated inflammatory pathways and how changes in the neuroendocrine-immune system are programmed. 

The importance of the adrenal gland in fetal physiology as a master regulator of fetal maturation and the initiation of parturition was first highlighted in the pioneering work of Liggins and colleagues in the 1960 s and 70 s [10]. More recently, publications have reported that sustained or repeated fetal stressors can activate the hypothalamic-pituitary-adrenocortical (HPA) axis resulting in elevated cortisol production and early maturation of the adrenal cortex contributing to fetal growth restriction. In their review, D. A. Myers and C. A. Ducsay have explored the impact of adrenocortical and adipose responses to high altitude-induced, long-term hypoxia in the ovine fetus. They show that adaptive responses both at the level of the hypothalamus and anterior pituitary as well as the adrenal cortex contribute to altered adrenal gland function that may be invoked as a general adaptive response to intrauterine stress to promote fetal survival. Given the importance of the HPA axis in regulating fetal maturation, glucocorticoids are administered to pregnant women at risk of preterm labour to promote fetal lung surfactant maturation with lifesaving consequences. A number of reviews in this issue examine the administration of antenatal glucocorticoids from a developmental programming perspective. L. Bennet et al. report that the overall impact of glucocorticoid administration on neurodevelopment is surprisingly unclear and, worryingly, that there is evidence of postnatal effects associated with impaired brain development following prenatal exposure to glucocorticoids. They discuss the possibility that maternal glucocorticoids may well be both protective and damaging, depending on the precise timing before the preterm fetus and infant are exposed to an hypoxia/ischemia insult. The authors conclude that, as with so many aspects of prenatal care and neonatology, timing is everything. Further examining the need for careful use of glucocorticoids, J. Morrison et al. review the impact of glucocorticoid use in the IUGR fetus and highlight the important observation that antenatal glucocorticoids may not have the same effects in IUGR preterm fetuses as they do in normally grown preterm fetuses.

Not only does this issue focus upon the direct impact of complications arising from a suboptimal *in utero* environment, we also include reviews dealing with how existing maternal conditions may impact the lifelong health trajectories of their offspring. Epidemiological studies have identified that maternal asthma, during pregnancy, increases the risk of a number of poor outcomes for the neonate including growth restriction, lower birth weight, preterm delivery, the need for neonatal resuscitation, and stillbirth. V. L. Clifton et al. discuss the evidence currently available to support the hypothesis that children of mothers with asthma may be at risk of lifelong health complications that include diabetes and hypertension. An understudied area of public health concern involves treatment of maternal conditions and their later life implications for the developing offspring. By way of example, there is considerable uncertainty regarding prescribing practices for pregnant women with severe and persistent psychiatric disorders. S. Raha et al. discuss the evidence for associating altered birth weight and maternal antipsychotic use, highlighting the role of the placenta and the possible impact of oxidative stress upon fetal growth. This review sets the stage for further studies to better our understanding of the effects of these drugs on fetal growth and consequences for the long-term health of the offspring.

Recently, studies have also begun to highlight how an adverse *in utero* environment may impact longevity. A key component of longevity is telomere health and a number of studies have now identified that telomere development may be impaired in association with altered fetal development, and the etiology of the diseases associated with the metabolic syndrome is beginning to be elucidated. In the review by S. E. Hallows et al. the authors suggest that premature aging or cellular senescence through increased oxidative stress and DNA damage to the telomeric ends of chromosomes may be initiators of disease processes. Finally, treatment at the extremes of the U-shaped birth weight distribution is limited and prevention of the increased risk of developing metabolic syndrome in later life is an area of active investigation. To this end, N. Ma and D. B. Hardy explore a number of the developmental processes contributing to the metabolic syndrome and discuss possible intervention strategies.

This collection of review articles highlights the wide and varied range of insults that the fetus may experience *in utero*, to which the fetus can respond through a range of adaptations which themselves may impact on the life course of that individual. A better understanding of these wide ranging insults and the similarities and differences in the fetal responses and adaptation will not only allow the prediction of the impact on health in adult life but also guide us in the development of effective intervention strategies.

*Timothy R. H. Regnault* *Timothy R. H. Regnault* *Mark J. Nijland* *Mark J. Nijland* *Helen Budge* *Helen Budge* *Janna L. Morrison* *Janna L. Morrison*
